# The effect of the severity of parental alcohol abuse on mental and behavioural disorders in children

**DOI:** 10.1007/s00787-018-1253-6

**Published:** 2018-11-14

**Authors:** Kirsimarja Raitasalo, Marja Holmila, Marke Jääskeläinen, Päivi Santalahti

**Affiliations:** 1 National Institute for Health and Welfare, Helsinki, Finland; 2 Social Psychology, University of Eastern Finland, Joensuu, Finland; 3 Sociology, University of Helsinki, Helsinki, Finland; 4 Child Psychiatry, University of Turku, Turku, Finland

**Keywords:** Alcohol abuse, Substance abuse, Parents, Family, Mental and behavioural disorders, Register data

## Abstract

Many studies have shown that children of alcohol abusing parents have a higher risk for mental and behavioural disorders compared to other children. Using a retrospective population-based cohort study, based on health care and social welfare registers that include children born in Finland in 1997 and their biological parents, we evaluated whether the severity of parental alcohol abuse is related to these disorders in children. We examined (1) differences in the incidence of mental and behavioural disorders over time among the children of parents with no alcohol problems, parents with less severe alcohol problems and parents with severe alcohol problems, and (2) associations between mother’s and father’s alcohol abuse and children’s risk of disorders. Children were followed up until the age of 15. A diagnosis of mental or behavioural disorders during the follow-up was received by 15.4% of the boys and 9.0% of the girls. Both less severe alcohol abuse (HR = 1.36, 95% CI 1.14–1.61) and severe alcohol abuse (HR = 1.29, 95% CI 1.11–1.49) in mothers increased the risk of these disorders in their offspring. The corresponding figures among fathers were HR = 1.19, 95% CI 0.92–1.54 and HR = 1.16, 95% CI 1.02–1.32. Our results indicate that both maternal alcohol abuse and paternal alcohol abuse, regardless of severity, are associated with an increased risk of mental and behavioural disorders in children. It is crucial for professionals working with children to pay attention to all children whose parents have any alcohol abuse problems.

**Electronic supplementary material:**

The online version of this article (10.1007/s00787-018-1253-6) contains supplementary material, which is available to authorized users.

## Introduction

Children in families where alcohol use dominates family life are particularly vulnerable. Parents’ alcohol abuse can lead to the parents having an insufficient ability to provide a safe environment for their children and to respond adequately to their children’s physical and emotional needs [1]. This in turn can lead to many further adverse consequences for children: parental alcohol abuse has been shown to be related to children’s cognitive, emotional and behavioural problems [2–4] and also to mental health problems later in adolescence and adulthood [5–7]. Parents’ alcohol abuse is also linked with other adversities in the family—like poverty, lack of education and mental health problems [8]—which can further complicate the children’s lives. The early and long-standing risks to children’s psychosocial health are important factors leading to health inequities in society [9, 10].

A Finnish study found that in substance abuse treatment contexts children of substance-abusing parents are seldom met in person, and their needs are rarely considered [11]. A similar observation has been made in Sweden [12]. In many countries there are not enough services focusing on these children, and the professionals meeting substance-using parents have not been trained to work with children [13]. Still, many kinds of services and professionals have an opportunity to meet the children of substance-using parents, for example nurses in maternity clinics, general practitioners, health care professionals working in educational institutions, teachers and social workers. Early intervention is an opportunity to offer support and keep a watching brief of children’s welfare, thus preventing problems from escalating [14]. One of the questions raised by the related literature is whether it is more efficient to focus on children whose parents are in the early stages of substance misuse rather than to focus on those whose parents already have serious substance abuse problems. It is also unclear whether the integrated approach of working with the whole family is more efficient than focusing on the children independently. These questions are linked to the question about the extent of harm experienced by children in the early stages of parental addiction or in families with temporary and less severe substance misuse and to the question about the extent of the most serious harm that is only experienced by children in families with the most severe problems.

Although many studies show the adverse effect of parental alcohol abuse on children [15], there is a lack of research on how the severity of parental problems is related to outcomes in children. However, there are several studies that show a strong linear relationship between parental psychiatric symptoms, such as depression and anxiety, and mental and behavioural problems in children [16, 17]. As similar effects on children have been found with regard to parental mental health and addiction problems, we hypothesize that the heavier the load of alcohol abuse in the parents, the greater the risk of negative outcomes, both for their children and for the parents themselves.

Using register-based data, we will explore whether the severity of parental alcohol abuse is related to other parental problems, such as long-term financial difficulties, mental health problems, low education level and not living with the child. This is used as a sensitivity analysis in order to verify the existence of a graded relationship between the severity of the alcohol problem and adverse outcomes. After that, we will examine how the severity of parents’ alcohol problems affects their children’s risk of mental and behavioural disorders in childhood.

## Data and methods

The data for the total birth cohort were used rather than only a sample in order to reach sufficient statistical power in studying the fairly infrequent cases of alcohol problems. The study used population-level data from national health care and social welfare registers [18, 19]. These registers were (1) the Medical Birth Register, (2) the Population Register, (3) the Care Register for Health Care, (4) the Care Register for Social Welfare, (5) the Register of Social Assistance, (6) the Register of Congenital Malformations, (7) the Prescription Register, (8) the Special Refund Entitlement Register, (9) the Register of Completed Education and Degrees and (10) Causes of Death Statistics. The data collection began with the Medical Birth Register, from which we obtained the personal identity numbers (assigned to all Finnish residents at birth or upon taking up residency) of all children born in 1997 (*N* = 59, 131) and their biological mothers. The personal identity numbers of fathers were obtained from the Population Register. We excluded children (and their parents) who had been given up for adoption (*n* = 131), who had migrated (*n* = 1288) or who had died (*n* = 333) during the follow-up. There were 799 children whose fathers were not registered in the records of the Population Register Centre, and thus their fathers could not be linked to the children. So, the final data consisted of 57,377 children, 57,074 mothers and 56,714 fathers. Data linkages were achieved via the personal identity numbers. Children and parents were followed from the child’s birth (in 1997) until the end of 2012.

Data collection, register linkages and anonymization of the data were carried out by the register keepers at the National Institute for Health and Welfare (THL), the Social Insurance Institution of Finland, and Statistics Finland. The Ethical Review Board of THL approved the study plan.

### Measurements

#### Parents

Parents with alcohol problems were identified using the Care Register for Health Care, the Care Register for Social Welfare, the Prescription Register, the Causes of Death Statistics and the Register of Congenital Malformations (for mothers only). Using the register data, it is impossible to measure the severity of alcohol problems as a continuous variable; therefore, the severity of the parental alcohol problem was classified into two categories. The parent was defined as having a less severe alcohol problem if he or she only had a primary or secondary ICD-10 diagnosis [20] related to acute drunkenness or harmful use (F10.0, F10.1), accidental alcohol poisoning (T51.0, X45) (excluding suicide attempts using alcohol among other things) or had purchased medication to treat alcohol abuse (the Anatomical Therapeutic Chemical Classification System [21] code N07BB) (this medication is usually prescribed by primary health care professionals). The parent was categorized as having a severe alcohol problem if he or she had a primary or secondary ICD-10 diagnosis of alcohol dependence or alcohol-induced psychosis (F10.2–9), an alcohol-related somatic disease (E24.4, G31.2, G40.51, G62.1, G72.1, I42.6, K29.2, K70.0–9, K85.2, K86.0, R78.0), another diagnosis of an external cause indicating a severe alcohol problem (O35.4, Z50.2, Z71.4, Z72.1, Y90.0–9, Y91.0–9), if she or he had been treated in a social welfare unit for alcohol abuse or if they had died from alcohol-related causes (excluding suicides).

Parents were classified as having psychiatric disorders if they had an ICD-10 diagnosis (either primary or secondary) or a record of inpatient treatment related to schizophrenia, schizotypal and delusional disorders (F20–29), mood disorders (F30–39), neurotic, stress-related and somatoform disorders (F40–48) and disorders of adult personality and behaviour (F60–69) in the Inpatient Health Care Register or the Care Register of Social Welfare.

As indicators of parents’ socio-demographic status, we used post-secondary school education, long-standing poverty and living with a child. Data on education at the end of the follow-up time were obtained from the Register of Completed Education and Degrees. This was classified dichotomously: (1) any recorded education after secondary school (upper-secondary school, vocational or university education) and (2) only basic education. Long-standing poverty was defined as having register entries in the Register of Social Assistance, entered upon having received social assistance for more than 3 months per year for at least 3 years. In order to detect whether the parents have lived with their children, we compared the building codes and the dates of moving in and out of a specific building using data from the Population Register. The parents were divided into two categories according to whether they had been living with their child or not for at least 1 year during the follow-up.

#### Children

Inpatient health care treatment and outpatient visits to public hospitals with two primary and four secondary ICD-10 diagnoses are registered in the Inpatient Health Care Register. We looked at register entries in four individual categories of mental and behavioural disorders in children: (1) mood disorders (F30–39, later F3), (2) neurotic, stress-related and somatoform disorders (F40–48, later F4), (3) disorders of psychological development (F80–89, later F8), and (4) behavioural and emotional disorders (F90–98, later F9). We also used any diagnosis of a mental and behavioural disorder as an outcome.

The class of diagnoses related to mood disorders (F3) contains disorders in which the fundamental disturbance is a change in affect or mood to depression (with or without associated anxiety) or to elation. Neurotic, stress-related and somatoform disorders (F4) are disorders in which anxiety is the predominant symptom. Disorders of psychological development (F8) include disturbances in the development of speech, language, the ability to learn, motor skills and social interaction. Common to disorders of this class is their early beginning and delays associated with the biological maturation of the central nervous system. Behavioural and emotional disorders (F9) include hyperkinetic disorders (e.g., attention-deficit hyperactivity disorder), behavioural and conduct disturbances that appear as asocial behaviour or aggressiveness and other behavioural and emotional disorders with onset usually occurring in childhood and adolescence.

### Statistical analysis

First, using cross-tabulation with the *χ*^2^ test and logistic regression analysis, we compared parents with both severe and less severe alcohol abuse to parents with no alcohol abuse in order to find out if alcohol problems were associated with other parental problems. Furthermore, using multivariate logistic regression, we compared the parents with severe alcohol abuse to parents with less severe alcohol abuse in order to detect possible differences between these two groups in the level of risk of having problems. In the adjusted models, we standardized the child’s gender, together with parental problems.

Second, Kaplan–Meier one minus survival functions with log rank (Mantel–Cox) tests were used to study the temporal incidence of mental and behavioural disorders among the children of parents with no alcohol problems, parents with less severe alcohol problems and parents with severe alcohol problems. These analyses were conducted for all categories of mental or behavioural disorders together. Finally, Cox proportional hazard models that used age as the time scale were fitted to evaluate associations between mothers’ and fathers’ alcohol abuse and risks of separate categories of disorders (F3, F4, F8, F9 and all categories together) in children, adjusting for potential confounders (parental education and the receipt of long-term social assistance, parents living with the child, parents’ psychiatric disorders and the child’s gender). These analyses were conducted separately for maternal and paternal effects. Adjusted hazard ratios (HRs) and 95% confidence intervals (CIs) are reported for each model. Analyses were conducted using the SAS 9.3 statistical package [22].

## Results

### Parents

Table 1 shows that among mothers with children born in 1997, 1.3% had at least one register entry related to severe alcohol abuse (according to our definition of it), and 1.0% had a register entry related to less severe alcohol abuse. Among fathers, the corresponding figures were 2.8% and 0.6%. The proportion of parents with less than 10 years of education was significantly higher among parents with alcohol abuse than among other parents. The same was true for having received social assistance, having a diagnosis of a psychiatric disorder and not living with the child. Furthermore, all problems were more prevalent among parents with severe alcohol abuse than among those with less severe alcohol abuse. When looking at the risk of parental problems by using multivariate logistic regression, we found some differences between mothers with less severe alcohol abuse and severe alcohol abuse. Mothers with severe alcohol abuse had a greater risk of financial difficulties (OR = 1.73, 95% CI 1.36−2.19), psychiatric disorders (OR 1.52, 95% CI 1.20−1.93) and not living with the child (OR = 2.99, 95% CI 1.48–6.02), compared to mothers with less severe alcohol abuse. Fathers with severe alcohol abuse also had a greater risk of not living with their child than those with less severe abuse problems (OR = 1.63, 95% CI 1.15–2.30).

**Table 1:** The mother’s alcohol abuse (*N* = 57,074) and father’s alcohol abuse (*N* = 55,310) according to background factors, % (*n*) and adjusted OR (less severe vs. severe) with 95% CI

Alcohol abuse	No	Less severe	Severe	*p* (chisq)	AOR^a^ (less severe vs. severe)
Mother (all)	97.7 (55,738)	1.0 (581)	1.3 (755)		
Education < 10 year	10.4 (5820)	29.1 (169)	34.6 (261)	< 0.0001	1.02 (0.80 − 1.32)
Social assistance	11.8 (6583)	48.7 (283)	65.0 (491)	< 0.0001	1.73 (1.36 − 2.19)
Psychiatric disorder	2.9 (1622)	29.3 (170)	42.1 (318)	< 0.0001	1.52 (1.20 − 1.93)
Not living with the child > 1 year	0.7 (404)	1.7 (10)	6.0 (45)	< 0.0001	2.99 (1.48 − 6.02)
Father (all)	96.6 (53,434)	0.6 (328)	2.8 (1548)		
Education < 10 year	16.2 (8508)	33.2 (107)	34.9 (534)	< 0.0001	0.98 (0.75 − 1.28)
Social assistance	11.1 (5955)	39.9 (131)	44.4 (687)	< 0.0001	1.10 (0.85 − 1.43)
Psychiatric disorder	2.1 (1141)	34.5 (113)	38.4 (594)	< 0.0001	1.15 (0.89 − 1.48)
Not living with the child > 1 year	4.7 (2531)	14.3 (47)	21.5 (333)	< 0.0001	1.63 (1.15 − 2.30)

^a^The models were adjusted by the child’s gender as well as all variables related to the parents’ socioeconomic status

### Mental and behavioural disorders in children

Some diagnosis of mental or behavioural disorders during the follow-up was received by 15.4% of boys and 9.0% of girls. The most prevalent individual categories of diagnoses were those related to behavioural and emotional disorders (F9; in 8.2% of boys and 4.3% of girls) and disorders of psychological development (F8; in 8.7% of boys and 3.4% of girls). Mood disorders (F3; in 1.1% of boys and 1.3% of girls) and neurotic, stress-related and somatoform disorders (F4; in 1.4% of boys and 1.7% of girls) were less prevalent.

When looking at the prevalence of these disorders (Table 2), we found that all of them were more prevalent among children with parents with alcohol abuse. To continue, the prevalence was somewhat higher among children with parents with severe alcohol abuse than with parents with less severe alcohol abuse.

**Table 2:** Mental and behavioural disorders in children aged 0–15 arranged by the mother’s alcohol abuse (*N* = 57,074) and the father’s alcohol abuse (*N* = 55,310), % (*n*)

Alcohol abuse	No	Less severe	Severe	*p* (chisq)
Mother				
Any F	11.8 (6601)	24.6 (143)	28.2 (213)	< 0.0001
F3	1.1 (631)	3.4 (20)	4.8 (36)	< 0.0001
F4	1.4 (801)	3.8 (22)	54.8 (36)	< 0.0001
F8	6.0 (3323)	11.2 (65)	11.8 (89)	< 0.0001
F9	6.0 (3349)	15.2 (88)	18.8 (142)	< 0.0001
Father				
Any F	11.7 (6239)	18.9 (62)	20.0 (309)	< 0.0001
F3	1.1 (590)	2.7 (9)	2.8 (43)	< 0.0001
F4	1.4 (757)	2.4 (8)	3.0 (479)	< 0.0001
F8	5.9 (3139)	8.5 (284)	9.8 (152)	< 0.0001
F9	5.9 (3154)	12.2 (40)	11.1 (172)	< 0.0001

**Fig. 1:**
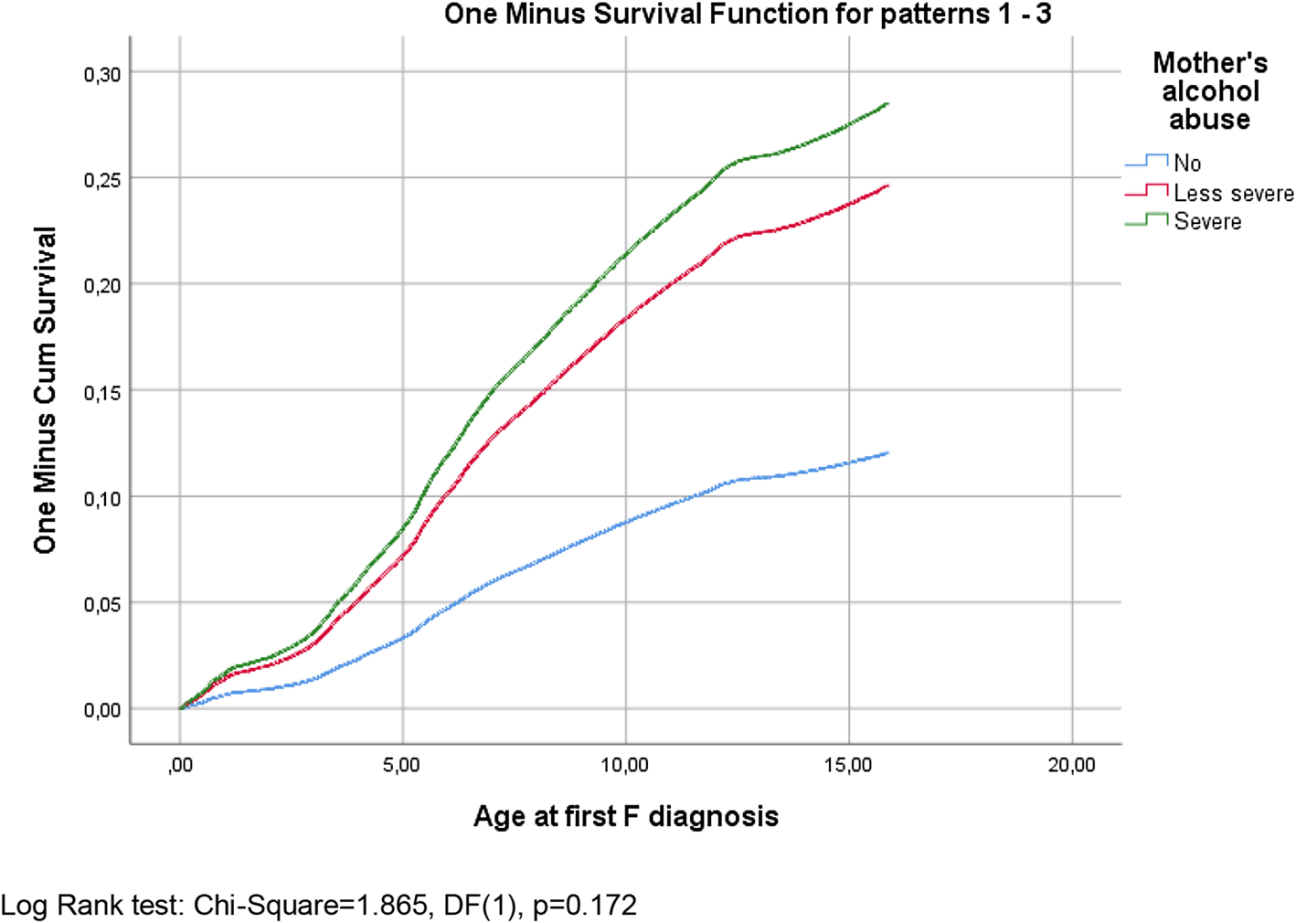
The Kaplan–Meier one minus survival functions for any mental or behavioural disorders in children displayed according to the mother’s alcohol abuse with the log rank (Mantel–Cox) test of the equality of survival distributions for any mental or behavioural disorders and the different levels of the mother’s alcohol abuse (less severe vs. severe)

**Fig. 2:**
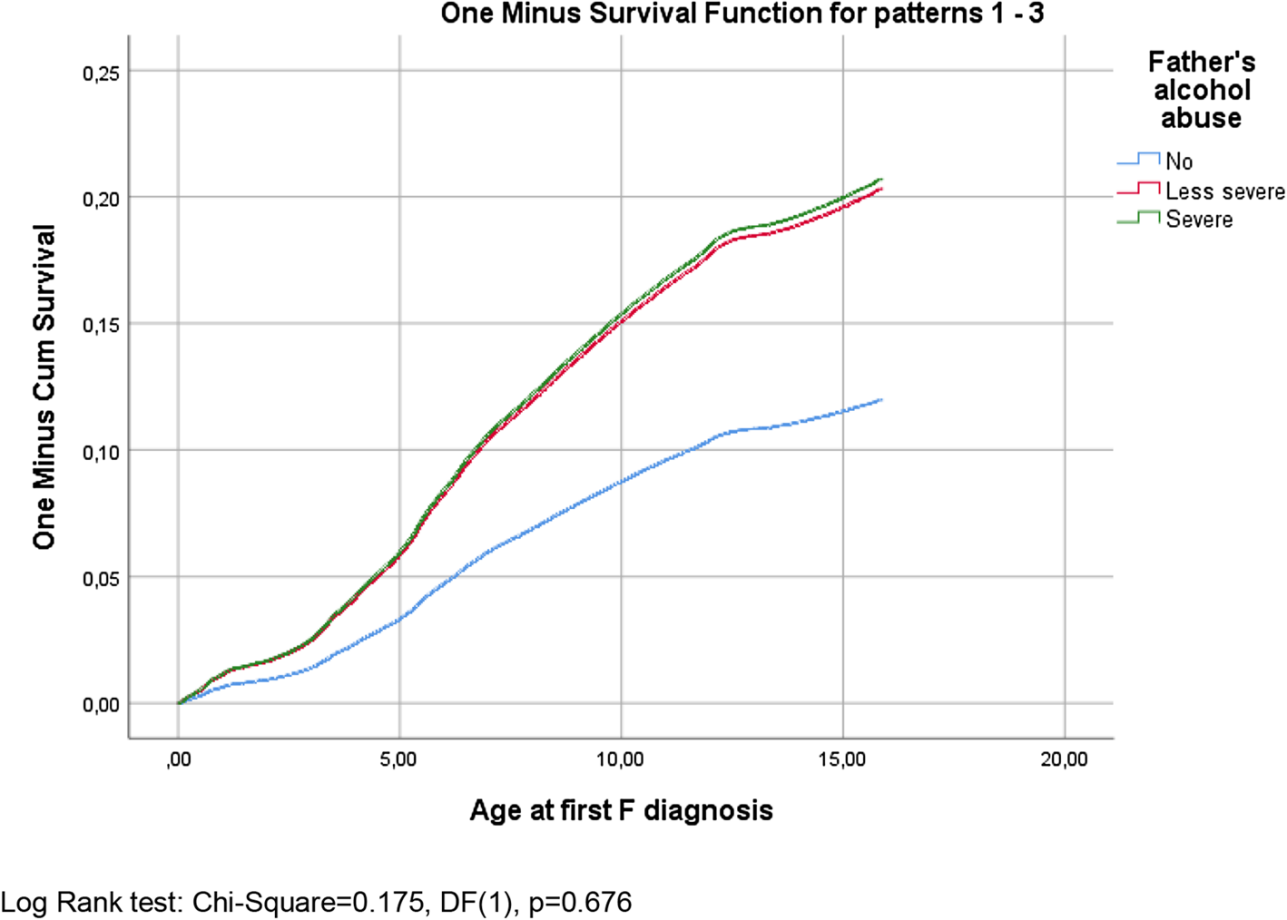
The Kaplan–Meier one minus survival functions for any mental or behavioural disorders in children displayed according to the father’s alcohol abuse with the log rank (Mantel–Cox) test of the equality of survival distributions for any mental or behavioural disorders and the different levels of the father’s alcohol abuse (less severe vs. severe)

The Kaplan–Meier (one minus) survival functions for any mental or behavioural disorders in children, displayed according to the mother’s and father’s alcohol abuse, are shown in Figs. 1 and 2. Based on the log rank (Mantel–Cox) test of the equality of survival distributions, both the mother’s and father’s less severe and severe alcohol abuse were associated significantly with their children’s incidence of mental and behavioural disorders (*p* < 0.000). However, the differences between groups in which there was less severe abuse and severe abuse were not significant (log rank tests: *χ*^2^ = 1.865, DF (1), *p *= 0.172 for mothers; *χ*^2^ = 0.175, DF (1), *p* = 0.676 for fathers). The same was true for all the separate categories of disorders (Supplementary Figs. 1–8, with test results).

Using Cox proportional hazard models, we standardized the effects of other parental problems and the child’s gender, in addition to alcohol abuse (Tables 3 and 4), when looking at the children’s risks of mental and behavioural disorders. This analysis shows that the risk of any mental or behavioural disorder in children was higher when the mother had alcohol abuse problems. The severity of the alcohol abuse did not make a difference. Among fathers only severe alcohol abuse increased the risk of any mental or behavioural disorders in children. When looking at the separate categories of disorders, we found similar patterns. The mother’s severe alcohol abuse increased the children’s risk of all categories of disorders except F8. Also, less severe alcohol abuse in mothers increased the risk of disorders in category F9. Among fathers, less severe but not severe alcohol abuse increased the risk of children’s disorders in category F9.

**Table 3:** Multivariable analysis of the association between mothers’ alcohol abuse and mental and behavioural disorders in separate ICD-10 categories in children aged 0–15

ICD-10	F	F3	F4	F8	F9
	HR	HR	HR	HR	HR
Mother LS^a^ (ref = no)	1.36 (1.14−1.61)	1.45 (0.91−2.30)	1.49 (0.96−2.31)	1.25 (0.97−1.63)	1.50 (1.20−1.87)
Mother S^b^ (ref = no)	1.29 (1.11−1.49)	1.51 (1.04−2.20)	1.51 (1.05−2.19)	1.11 (0.88−1.39)	1.51 (1.26−1.82)
Mother’s educ. (ref = no)	0.82 (0.76−0.88)	0.87 (0.71−1.08)	0.96 (0.79−1.17)	0.77 (0.70−0.85)	0.89 (0.81−0.97)
Social assist. m (ref = no)	2.03 (1.91−2.16)	2.65 (2.21−3.17)	2.07 (1.75−2.46)	1.94 (1.78−2.11)	2.33 (2.15−2.53)
Mother’s psyc. (ref = no)	1.66 (1.51−1.83)	2.43 (1.89−3.13)	2.16 (1.70−2.76)	1.43 (1.24−1.65)	1.78 (1.57−2.02)
Living w. mother (ref = no)	0.93 (0.74−1.16)	0.90 (0.49−1.64)	0.97 (0.53−1.77)	0.82 (0.61−1.01)	0.99 (0.73−1.34)
Child’s gender (ref = boy)	0.56 (0.53−0.59)	1.21 (1.04−1.40)	1.19 (1.04−1.36)	0.38 (0.35−0.41)	0.51 (0.48−0.55)

*HR*  hazard ratio, *95% CI* 95% confidence interval

^a^Less severe alcohol problem

^b^Severe alcohol problem

**Table 4:** Multivariable analysis of the association between fathers’ alcohol abuse and mental and behavioural disorders in separate ICD-10 categories in children aged 0−15

ICD-10	F	F3	F4	F8	F9
	HR	HR	HR	HR	HR
Father LS^a^ (ref = no)	1.19 (0.92−1.54)	1.56 (0.80−3.06)	1.13 (0.56−2.29)	1.10 (0.75−1.60)	1.39 (1.01−1.92)
Father S^b^ (ref = no)	1.16 (1.02−1.32)	1.33 (0.93−1.90)	1.20 (0.85−1.68)	1.18 (0.98−1.42)	1.13 (0.95−1.35)
Father’s educ. (ref = no)	0.82 (0.77−0.87)	0.95 (0.78−1.16)	0.84 (0.71−1.00)	0.79 (0.72−0.86)	0.84 (0.77−0.91)
Social assist. f (ref = no)	1.66 (1.56−1.78)	2.45 (2.02−2.97)	1.78 (1.48−2.13)	1.66 (1.52−1.82)	1.85 (1.70−2.02)
Father’s psyc. (ref = no)	1.32 (1.17−1.48)	1.42 (1.02, 1.98)	1.53 (1.13−2.08)	1.20 (1.01−1.43)	1.33 (1.13−1.55)
Living w. father (ref = no)	0.66 (0.60−0.72)	0.62 (0.48−0.80)	0.59 (0.47−0.74)	0.76 (0.66−0.86)	0.56 (0.50−0.62)
Child’s gender (ref = boy)	0.56 (0.53−0.59)	1.24 (1.06−1.45)	1.22 (1.06−1.40)	0.38 (0.35−0.41)	0.51 (0.48−0.55)

*HR* hazard ratio, *95% CI* 95% confidence interval

^a^Less severe alcohol problem

^b^Severe alcohol problem

The effects of other parental problems on children’s disorders were mixed. Both the mother’s and father’s education after secondary school decreased the children’s risk of any disorder. Among both mothers and fathers, education decreased the risk of F8 and F9 in their children. The mother’s and father’s receipt of long-term social assistance increased the children’s risk of all studied categories of disorders. Also psychiatric disorders in both mothers and fathers increased the children’s risk of all categories of disorders. Living with the mother was not related to children’s risk for any of the studied categories of disorders, but living with the father decreased the risk of all categories of disorders. Girls had a higher risk than boys of disorders of categories F3 and F4, and a lower risk of disorders of categories F8 and F9.

## Discussion

Our results indicate that parents’ alcohol abuse has a negative effect on children’s psychological well-being, regardless of the severity of the problem or other psychiatric disorders, the parents’ level of education, financial difficulties or living arrangements. Both mothers’ and fathers’ alcohol abuse was related to mental and behavioural disorders in children, although the mother’s alcohol abuse had a more harmful effect than that of the father’s.

Our study extends the existing literature, suggesting important links between parental alcohol abuse and harm to children. The positive association between parental alcohol abuse and mental and behavioural disorders in children corresponds with the results of previous studies on this topic [2–4].

The difference in the effects of the mother’s and father’s alcohol abuse was in accordance with previous research [23, 24]. According to a previous study using the same data, both parents’ alcohol abuse has even stronger effect on mental and behavioural disorders in children than when only one parent has alcohol abuse problems. This indicates that also father’s alcohol abuse has an independent effect regardless of mother’s alcohol abuse [25]. Some of the differences between the effects of maternal and paternal alcohol abuse may be explained by the fact that alcohol abusing fathers do not live with their children as often as do alcohol abusing mothers [25]. Although family separation has been found to be a risk factor for mental and behavioural disorders in children [25–27], not living with the alcohol abusing parent is likely to protect the child against the harmful effects of parental alcohol abuse. Moreover, women (and mothers) with substance use disorders have been found to be more likely than men to have psychiatric disorders such as depression, anxiety, eating disorders and lower self-esteem and to have a history of victimisation, homelessness and to have experienced violence [28, 29]. This accumulation of problems can be one explanation for the higher risk of mental and behavioural disorders in the children of alcohol abusing mothers compared with children of fathers with these problems. Our results thus emphasize the mother’s role in children’s well-being in our culture. This is perhaps because the daily care of children still tends to be seen as the mother’s main responsibility rather than the father’s, even if families differ in this.

Furthermore, alcohol abuse during pregnancy is also a well-known risk factor for the outcomes of this study. Children exposed to maternal alcohol use during pregnancy have more problems related to cognitive and psychosocial development [30] and mental health [31] than other children. Extensive research on the effects of prenatal alcohol exposure supports the existence of a spectrum of diagnostic conditions, collectively referred to as fetal alcohol spectrum disorders (FASD) [32]. Individuals diagnosed with FASD often have neurodevelopmental disabilities such as neurocognitive impairment, impairment in self-regulation and deficits in adaptive functioning, which largely overlap with many diagnoses of mental and behavioural disorders [33]. However, it is not possible to separate the effect of prenatal alcohol exposure with our data as the register entries only detect the timing of treatment or death and not the timing of alcohol abuse.

Our results offer new information on how the severity of parental alcohol problems is related to negative outcomes in the mental health of children. The severity of alcohol abuse in either mothers or fathers did not make a difference in the risk of mental or behavioural disorders in their children.

The main strength of our study is that register data offer an exceptional possibility to study entire cohorts and otherwise hard-to-reach populations and difficult phenomena at low costs and without the problems of response rates. Even though not very detailed, the data in registers are based on evaluations and diagnoses made by professionals, which eliminates social desirability bias.

The limitations of our study are mainly related to the underrepresentation of the measured phenomena in the register data. Some parents with alcohol abuse may not be represented because they have not used the services included in the registers. Only a small fraction of alcohol abusers in the general population end up in registers [34]. Our data thus reaches only the ‘tip of the iceberg’ of the phenomenon, often noted in literature; registers do not include data on occasional use or abuse of alcohol or on patients within primary health care [18]. Thus, we do not know whether the effect of parents’ risky alcohol use (which has not necessarily yet developed as a problem) on their children is similar to the effects in this study [23].

According to our definition of severity, there were more parents with severe than with less severe alcohol abuse. We can assume that all cases ending up in registers are somewhat severe, as we know that most people with alcohol problems never end up in care and thus are not in the registers [34]. In our data, parents with less severe alcohol abuse were represented by those who only had a register entry for alcohol use or those who had purchased prescription medication for treating alcohol abuse (often prescribed by primary or occupational health care professionals) and had not been treated in health or social care units or died because of alcohol abuse. We can nonetheless assume that also they represented the higher end of the spectrum of alcohol use and abuse. However, our sensitivity analysis on parental problems related to the severity of the alcohol abuse indicated that our definition separates the severe and less severe cases of alcohol abuse. Even though the parents with less severe alcohol abuse encountered less problems than parents with severe alcohol abuse, their children had similar risks of mental and behavioural disorders. It is likely that ‘a threshold’ for these risks is realised on the lower levels of alcohol abuse that we were able to capture with register data.

It should also be noted as a limitation that the use of a clinical diagnosis or the purchase of a prescription drug as indicators of alcohol abuse may mean that the reference category of no abuse may still contain alcohol abusing parents. Furthermore, it may take several years from the onset of alcohol abuse to seeking of treatment [35], and thus we were not able to determine the onset of parental alcohol abuse or the actual duration of exposure to an alcohol-abusing parent.

One more limitation is that it was not possible to control for all relevant sociodemographic factors, such as the parents’ employment status and the region of the country (urban vs. rural) in our data. Moreover, as we only had data on biological parents, we do not know whether the child was living in a family or not where a social parent, such as the mother’s or father’s new spouse, abuses alcohol. Finally, with the administrative register data, we were not able to examine familial dysfunctions, such as various kinds of child maltreatment, that also adversely affect children [36]. Previous research has indicated that children growing up in families with parental alcohol abuse have a higher risk of emotional, physical and sexual abuse [37], which probably is one of the mediating mechanisms between parental alcohol abuse and children’s adverse mental health outcomes.

Regarding the clinical management of non-dependent high-risk drinkers, the cumulative evidence shows that brief interventions provided by health care professionals can produce clinically significant reductions in drinking and alcohol-related problems [38, 39]. Research evidence also suggests that the effect of changes in the retail availability of alcohol, including reductions in the hours and days of sale, limits on the number of alcohol outlets and restrictions on retail access to alcohol apply to all groups of drinkers, including heavy and problem drinkers [40]. The theory of collectivity in drinking culture [41, 42] suggests that as the per capita consumption in a population increases, the consumption of the heaviest drinkers also rises, as does the prevalence of heavy drinkers and the rate of alcohol-related harm. Along with this, alcohol’s harm to others, including children, can also be supposed to increase. In order to prevent the problems for children caused by parents’ alcohol abuse, it is important to target interventions to the whole population.

In addition to aiming for reducing alcohol consumption in the entire population, interventions targeted at parents with children in all age categories are important in preventing alcohol’s harm to children. Psychological and/or educational interventions for reducing alcohol consumption have been shown to result in increased abstinence from alcohol and a reduction in alcohol consumption among pregnant women [43]. Psychosocial interventions aimed at substance-abusing mothers have also resulted in positive effects on child-related outcomes, on mothers’ abstinence and mental health and on parenting attitudes and behaviour [44]. Furthermore, longer school-, community- and family-based interventions that include children’s, parenting and family skills training components have been shown to have a positive effect on knowledge, coping skills, social behaviour, self-esteem, family functioning, and externalizing and internalizing the symptoms of the child [45, 46]. Preventive interventions for mentally ill, including substance-abusing, parents have also been shown to remarkably decrease the risk for new diagnoses of mental or behavioural disorders in children [47].

The treatment services for patients with any stage of alcohol abuse should be developed with the aim of helping the whole family. Timely and well-realised interventions could help in finding courses of action where authorities, health care professionals and the parents make the best decisions together concerning the child’s life [48]. Schools and day-care centres are important not only in recognising children’s problems but also in providing support and directing parents to specialised services. Also, services for adults should take responsibility for patients’ children in order to prevent the children from developing problems and to build cross-sectoral community-based services for families with multiple needs.

Many studies show that in order for children and adolescents to benefit from the dissemination and implementation of evidence-based practices, issues like fidelity monitoring and supervision have to be taken care of [49]. Most of the intervention studies are conducted in the USA, but it is likely that the principles of these interventions also apply in other countries, as has been found in other areas of psychosocial interventions on children’s mental health [50]. According to a US study, children with psychiatric symptoms of psychologically ill parents get less treatment than those with healthy parents [51]. Parental problems can thus increase not only the child’s risk of disorders but also his or her risk of being left without help. A growing body of research also suggests that there are often particular difficulties in gaining access to families impacted by chronic parental alcohol misuse [52], such as attitudes and beliefs about mental health and substance abuse treatment, inadequacies in services, children’s ambivalence about treatment and parental disagreement and lack of involvement [53–55]. In Finland, the children of parents with substance abuse or psychiatric disorders receive treatment relatively late, years after the first symptoms of disorders have occurred [56]. This means that disorders in children that are possibly related to parental alcohol abuse are not treated this early (when the child is aged 0–15) and thus do not come out in registers.

In the future, the effect of parental alcohol abuse on psychological and behavioural disorders in children should be studied in non-clinical populations (i.e., people without a clinical diagnosis or treatment contact) to see whether the relationship also exists in cases where the alcohol abuser has not sought help for the problem. It is also important to focus on possible buffering factors that protect the child from the adverse effects of parental alcohol abuse.

In summary, children with alcohol-abusing parents have a higher risk of mental and behavioural disorders regardless of the severity of parental alcohol abuse. Our results indicate that the early recognition of the family’s situation is crucial in preventing later problems in children’s lives.

## Electronic supplementary material

Below is the link to the electronic supplementary material.

Supplementary material 1 (DOC 218 kb)
