# Supplementary material for: The effect of the severity of parental alcohol abuse on mental and behavioural disorders in children
Source: Eur Child Adolesc Psychiatry. 2018 Nov 14;28(7):913–22. doi: 10.1007/s00787-018-1253-6 (PMC6647416; doi:10.1007/s00787-018-1253-6)
Supplement: Supplementary file 1 — Supplementary material 1 (DOC 218 kb) [file 787_2018_1253_MOESM1_ESM.doc]

Supplementary figure 1. The Kaplan–Meier one minus survival functions for mood disorders (F3) in children according to mother’s alcohol abuse with Log Rank (Mantel-Cox) test of equality of survival distributions for mood disorders (F3) for the different levels of mother's alcohol abuse (less severe vs. severe).


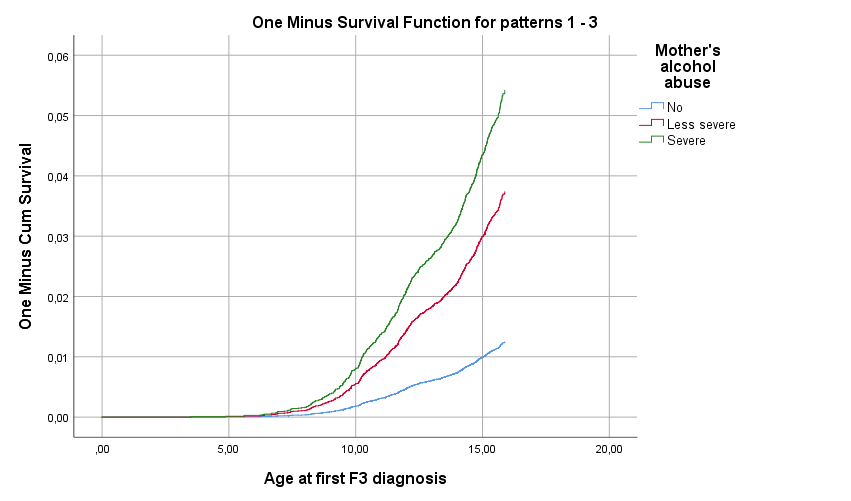


Log Rank test: Chi-Square=1.337, DF(1), p=0.248

Supplementary figure 2. The Kaplan–Meier one minus survival functions for mood disorders (F3) in children according to father’s alcohol abuse with Log Rank (Mantel-Cox) test of equality of survival distributions for mood disorders (F3) for the different levels of father's alcohol abuse (less severe vs. severe).


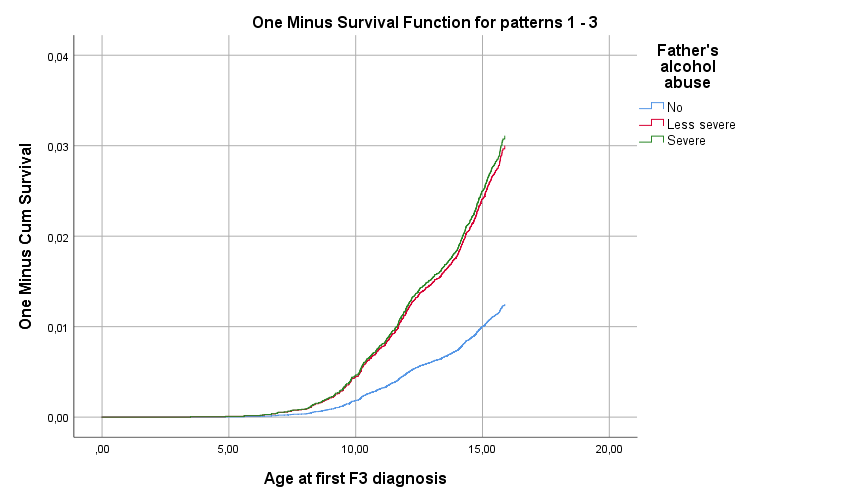


Log Rank test: Chi-Square=0.001, DF(1), p=0.973

Supplementary figure 3. The Kaplan–Meier one minus survival functions for mood disorders (F4) in children according to mother’s alcohol abuse with Log Rank (Mantel-Cox) test of equality of survival distributions for mood disorders (F4) for the different levels of mother's alcohol abuse (less severe vs. severe).


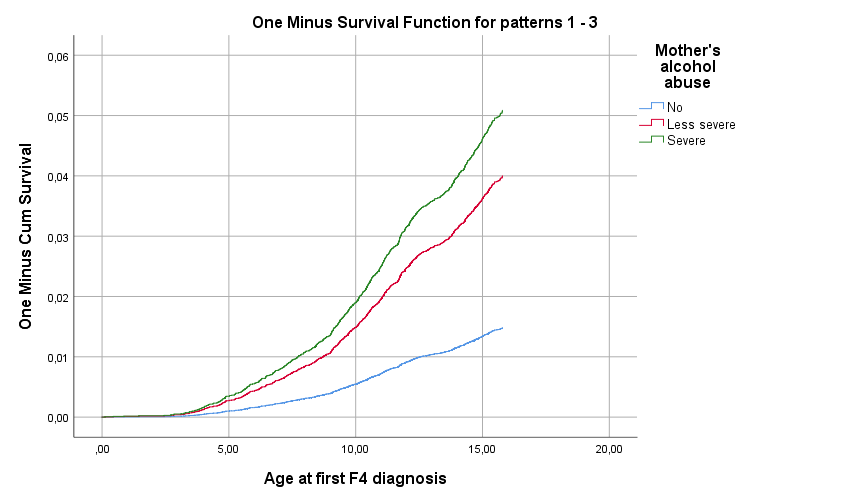


Log Rank test: Chi-Square=0.653, DF(1), p=0.419

Supplementary figure 4. The Kaplan–Meier one minus survival functions for mood disorders (F4) in children according to father’s alcohol abuse with Log Rank (Mantel-Cox) test of equality of survival distributions for mood disorders (F4) for the different levels of father's alcohol abuse (less severe vs. severe).


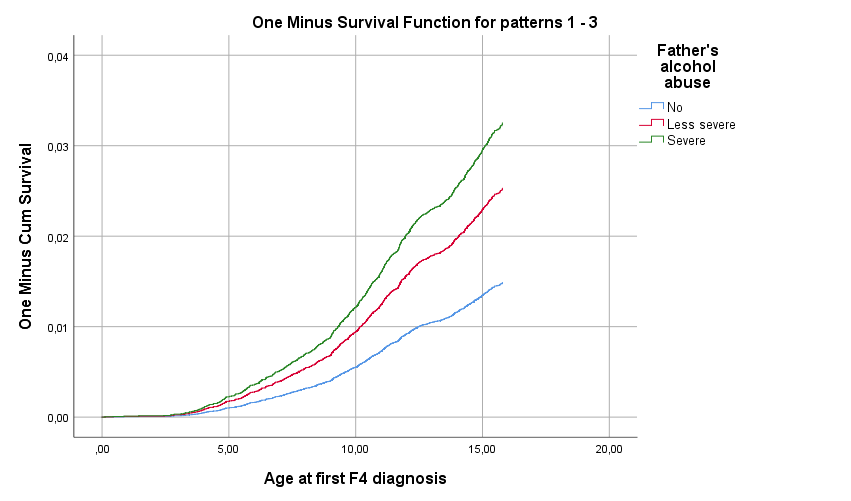


Log Rank test: Chi-Square=0.314, DF(1), p=0.575

Supplementary figure 5. The Kaplan–Meier one minus survival functions for mood disorders (F8) in children according to mother’s alcohol abuse with Log Rank (Mantel-Cox) test of equality of survival distributions for mood disorders (F8) for the different levels of mother's alcohol abuse (less severe vs. severe).


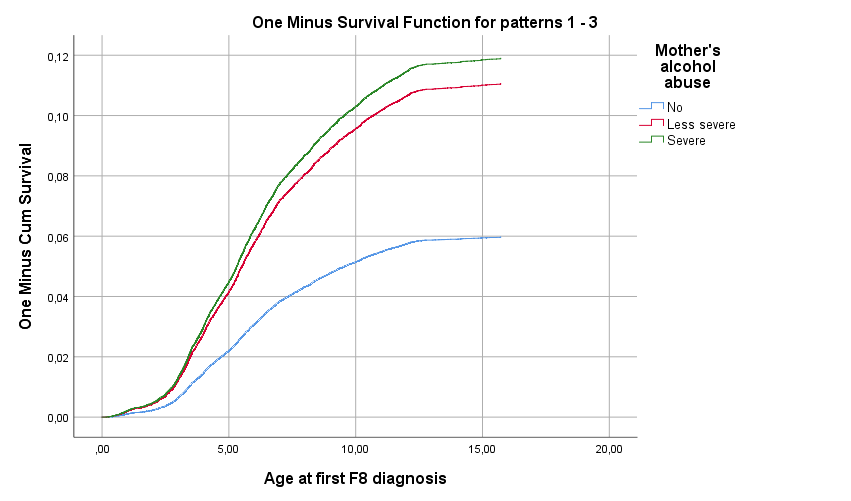


Log Rank test: Chi-Square=0.115, DF(1), p=0.734

Supplementary figure 6. The Kaplan–Meier one minus survival functions for mood disorders (F8) in children according to father’s alcohol abuse with Log Rank (Mantel-Cox) test of equality of survival distributions for mood disorders (F8) for the different levels of father's alcohol abuse (less severe vs. severe).


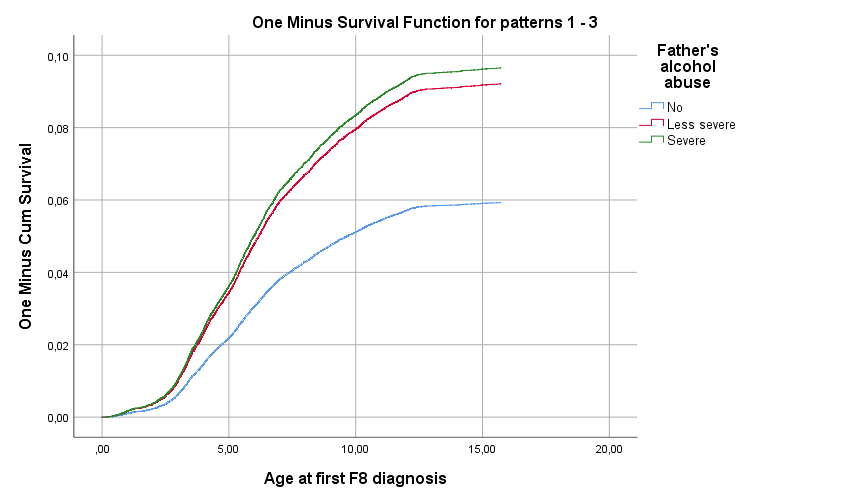


Log Rank test: Chi-Square=0.510, DF(1), p=0.475

Supplementary figure 7. The Kaplan–Meier one minus survival functions for mood disorders (F9) in children according to mother’s alcohol abuse with Log Rank (Mantel-Cox) test of equality of survival distributions for mood disorders (F9) for the different levels of mother's alcohol abuse (less severe vs. severe).


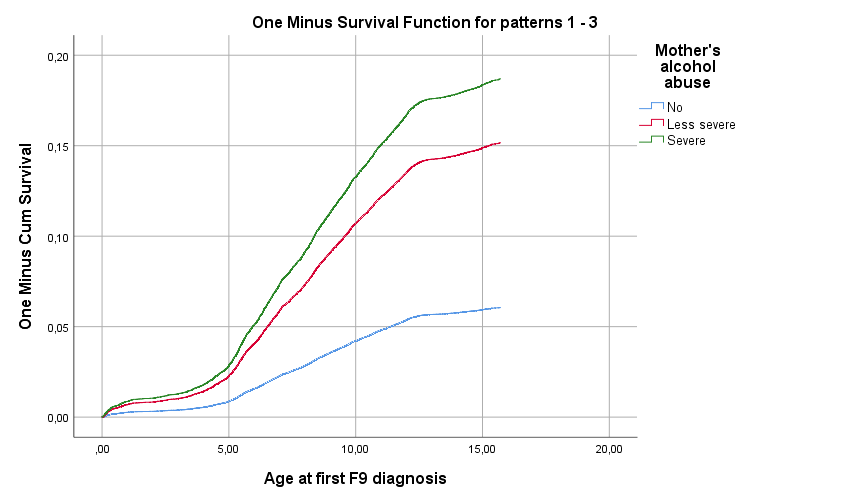


Log Rank test: Chi-Square=2.814, DF(1), p=0.093

Supplementary figure 8. The Kaplan–Meier one minus survival functions for mood disorders (F9) in children according to father’s alcohol abuse with Log Rank (Mantel-Cox) test of equality of survival distributions for mood disorders (F9) for the different levels of father's alcohol abuse (less severe vs. severe).


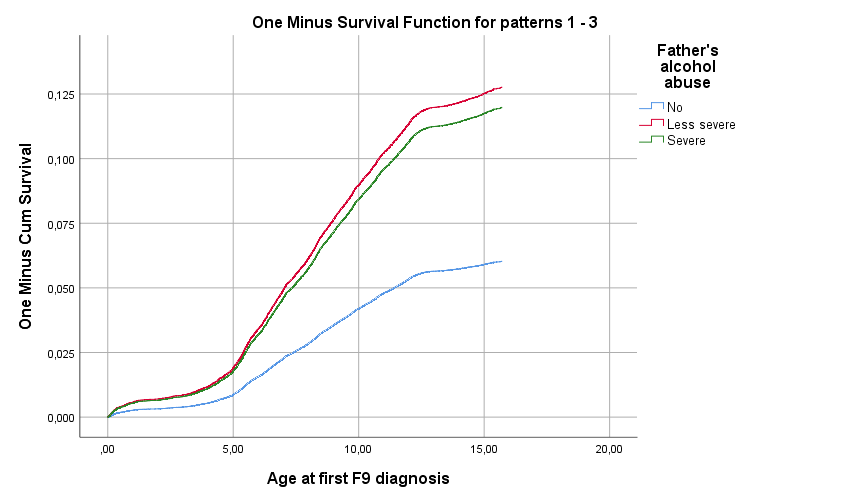


Log Rank test: Chi-Square=0.362, DF(1), p=0.547
